# Comparison of High n-3 PUFA Levels and Cyclic Heat Stress Effects on Carcass Characteristics, Meat Quality, and Oxidative Stability of Breast Meat of Broilers Fed Low- and High-Antioxidant Diets

**DOI:** 10.3390/ani14223314

**Published:** 2024-11-18

**Authors:** Manca Pečjak Pal, Jakob Leskovec, Alenka Levart, Tatjana Pirman, Janez Salobir, Vida Rezar

**Affiliations:** 1 Department of Animal Science, Biotechnical Faculty, University of Ljubljana, 1000 Ljubljana, Slovenia; jakob.leskovec@irta.cat (J.L.); alenka.levart@bf.uni-lj.si (A.L.); tatjana.pirman@bf.uni-lj.si (T.P.); janez.salobir@bf.uni-lj.si (J.S.); vida.rezar@bf.uni-lj.si (V.R.); 2 Animal Nutrition, Institute for Food and Agricultural Research and Technology (IRTA), 43006 Tarragona, Spain

**Keywords:** broilers, n-3 PUFAs, cyclic heat stress, antioxidants, oxidative stress, meat quality, meat oxidative stability

## Abstract

This study investigated the individual and combined effects of a high dietary n-3 PUFA intake and cyclic heat stress (HS) on the carcass characteristics, meat quality, and oxidative stability of broiler breast meat and the potential of antioxidant supplementation (vitamins E, C, and selenium) to mitigate these effects. A total of 192 one-day-old male Ross 308 broilers were randomly assigned to 24 pens within two controlled environment chambers and fed with the following diets: a basal diet low in antioxidants according to NRC recommendations (NRC group), a basal diet according to Aviagen recommendations additionally supplemented with 200 IU/kg vitamin E, 250 mg/kg vitamin C, and 0.15 mg/kg selenium (HAOX group), and these two diets further supplemented with 5% linseed oil (NRC N-3 and HAOX N-3 groups). On day 22, the broilers were exposed to the following two environmental conditions: thermoneutral (TN, 21 °C) or cyclic HS (HS, 34 ± 1 °C, 7 h/d) in a 2 × 2 × 2 factorial design. A high intake of n-3 PUFAs significantly decreased growth performance, dressing percentage, and breast yield, while the incidence of pale, soft, and exudative (PSE) meat characteristics and malondialdehyde (MDA) levels increased. Cyclic HS reduced body weight (BW) and average daily feed intake (ADFI), but had limited effects on meat quality. No interactions between n-3 PUFAs and HS were observed for any measurements. High antioxidant supplementation increased breast yield, improved meat quality, and reduced oxidative stress, as evidenced by an enhanced antioxidant activity and lower MDA levels. In conclusion, n-3 PUFAs had a negative effect on both the carcass characteristics and meat quality of broilers, while HS primarily affected only carcass characteristics, with neither stressor having severe adverse effects. High levels of antioxidants could mitigate the negative effects of dietary- and heat-induced oxidative stress by enhancing the oxidative stability of broiler meat.

## Simple Summary

Broiler chickens are often exposed to various nutritional and environmental stressors that negatively impact their productivity and overall health. These stressors, such as oxidized diets and high ambient temperatures, increase the risk of oxidative stress, which can affect meat quality and increase production costs. While the harmful effects of high n-3 polyunsaturated fatty acid (PUFA) levels and heat stress on broiler meat quality parameters have been documented, less is known about the combined effects of both these stressors. Antioxidant supplementation could play a key role in improving antioxidant defense and preventing oxidative damage to meat. Our study confirmed that dietary enrichment with 5% linseed oil and cyclic heat stress negatively affected the quality and oxidative stability of broiler breast meat, although no interactions between the stressors were found. In addition, high levels of antioxidants were found to improve meat quality and prevent oxidative damage to broiler meat.

## 1. Introduction

The global consumption of broiler meat is steadily increasing, as it provides an affordable and high-quality source of protein to maintain consumer nutritional and economic security [1]. However, intensive poultry production of the modern broiler genotype is often associated with various external stressors that lead to an increased production of reactive oxygen species (ROS) and alter the antioxidant defense system, which, in turn, causes oxidative stress [2]. Oxidative stress causes oxidative damage to major biological macromolecules, leads to a reduced production performance and health problems, and has negative effects on oxidative stability, as well as the sensory and nutritional quality of broiler meat [3].

To promote a better growth performance, chicken feed is usually enriched with plant oils, which are an energy-rich source of essential fatty acids. The enrichment of broiler feed with n-3 polyunsaturated fatty acids (PUFAs) is beneficial for enhancing the nutritional value of meat products and promoting animal and consumer health [4]. For the production of functional foods by improving the concentration of long-chain n-3 PUFAs in broiler meat, altering the fatty acid profile of broiler diets, which is highly associated with the incorporation of fatty acids into the muscle tissues of broilers, is essential [5]. However, chicken meat enriched with high levels of linseed oil, which is a good source of n-3 PUFAs, especially α-linolenic acid (LNA), is more susceptible to lipid oxidation due to the higher degree of unsaturation [6]. This can affect the quality of meat by deteriorating its taste, producing an unpleasant odor, and affecting its texture, flavor, color, and appearance [7]. Lipid oxidation also leads to the loss of fat-soluble vitamins and essential fatty acids, further reducing the nutritional value of the meat [8]. According to Betti et al. [6], feeding high levels of linseed oil to broilers for a prolonged period of time affected the functional properties (a lower ultimate pH and increased cooking loss, drip loss, and shear force) and color characteristics of broiler breast meat. High dietary levels of linseed oil have also been associated with an increased susceptibility of breast meat to oxidation, as indicated by elevated malondialdehyde (MDA) levels [6,9,10].

Broilers are particularly susceptible to heat stress (HS) due to their anatomical characteristics and genetic selection for rapid growth and larger breast muscles. Additionally, modern broiler genotypes have a high metabolic activity and produce more body heat, making it difficult for them to dissipate excess heat to the environment [11]. The exposure of broilers to HS has been reported to negatively affect their growth performance, reduce carcass traits, and impair meat quality and nutritional value by altering the deposition of protein and intramuscular fat in the muscles [12]. In addition, HS causes a rapid drop in muscle pH after slaughter, accelerating postmortem glycogenesis. This leads to an increased drip loss, cooking loss, lightness, and tenderness and a reduced juiciness of the meat, as well as an increased shear force of breast and thigh muscles [13,14,15].

Dietary supplementation of natural and/or synthetic antioxidants has been shown to mitigate the adverse effects of oxidative stress on oxidative stability and meat quality in broilers [16]. The most commonly used antioxidants in broiler diets are vitamins E and C and selenium, which are known to act synergistically to prevent oxidative damage when supplemented together [17]. Vitamin E is the most important fat-soluble antioxidant, which protects cells and tissues from lipid oxidation caused by free radicals. However, when vitamin E reacts with lipid radicals, it is oxidized and regenerated into its active form through the reduction of vitamin C, which acts as a co-antioxidant [18]. Selenium, an essential trace element for animals, plays an important role in enhancing the antioxidant defense system by supporting selenoproteins such as glutathione peroxidase (GPx) and thioredoxin reductase, which contribute to the removal of ROS. Selenium also plays an important role in the absorption of vitamin E and restores vitamin C activity [19]. Previous studies have already confirmed positive effects on the oxidative stability of meat under oxidative stress conditions when broiler diets were supplemented with a combination of vitamin E and selenium [20], vitamins E, C, and alpha-lipoic acid [21], and vitamins E, C, and selenium [22,23].

To our knowledge, no studies have investigated the combined effects of high n-3 PUFA levels and HS together with high antioxidant levels on the meat quality and oxidative stability of broiler meat. Therefore, the main objective of the present study was to investigate the individual and combined effects of a high intake of n-3 PUFAs and cyclic HS on the carcass characteristics, meat quality, and oxidative stability of broiler breast meat. Additionally, we aimed to determine whether high dietary supplementation with a mixture of vitamins E, C, and selenium is essential for mitigating oxidation processes and enhancing the oxidative stability of broiler breast meat under dietary- and heat-induced oxidative stress.

## 2. Materials and Methods

All animal procedures applied in this study were reviewed and approved by the Animal Ethics Committee of the Veterinary Administration of the Republic of Slovenia (project license number U34401-5/2021/4). The present study was performed at the research facility of the Department of Animal Science, Biotechnical Faculty, University of Ljubljana, Slovenia. The current trial was conducted as part of a larger research initiative in which broiler chickens were exposed to various forms of induced oxidative stress. Previous results on gut fermentation and mucosal morphology from this larger experiment were published by Rezar et al. [24].

### 2.1. Animals, Housing, and Dietary Treatments

A total of 192 one-day-old male Ross 308 broiler chickens were randomly allocated to two separate experimental rooms within the same experimental facility, as follows: a thermoneutral (TN) room and a heat stress (HS) room, each containing 12 deep litter pens. In each room, 96 birds were divided into four experimental groups based on dietary treatment (24 birds per group), with three replicates per group. This arrangement resulted in four dietary treatments (2 × 2 diets) and two environmental conditions in a 2 × 2 × 2 factorial design within randomized complete blocks. Each pen, measuring 0.95 m × 1.26 m (total 1.20 m^2^), was filled with wood shavings as litter and equipped with a plastic feeder and five nipple drinkers. Diet and water were provided ad libitum throughout the trial. The broilers were reared according to a standard lighting program that provided 23 h of light and 1 h of darkness during the first week and was changed to 18 h of light and 6 h of darkness from day 8 until the end of the experiment.

The experimental diets were formulated and administered in the following three phases: a starter diet for the first 10 days, a grower diet from day 11 to day 24, and a finisher diet from day 25 to the end of the experiment on day 42. The diets were based on either the National Research Council (NRC) minimal nutrient requirements [25] (low antioxidants) or the Aviagen recommendations for Ross 308 broilers [26] additionally supplemented with 200 IU vitamin E, 250 mg vitamin C, and 0.15 mg Se/kg (high antioxidants). The primary fat source in the experimental diets consisted of either a mixture of animal fats and plant oils or 5% cold-pressed linseed oil, resulting in n-3 PUFA-enriched diets. Accordingly, the four following dietary treatments were used in the experiment: a basal diet according to the NRC without additional supplementation (NRC group); a basal diet according to Aviagen + 200 IU dl-α-tocopheryl acetate + 250 mg vitamin C + 0.15 mg Se/kg diet (HAOX group); NRC + 5% linseed oil (NRC N-3 group); and HAOX + 5% linseed oil (HAOX N-3 group). Detailed insights into the composition and calculated energy and nutrient contents of the starter, grower, and finisher diet formulations are presented in Table 1. In addition, samples of the finisher diets were collected to determine the proximate composition and selenium content according to the standard methods of Naumann and Bassler [27]. Further analyses were performed to determine the composition of α-, γ + β, and δ -tocopherol, the antioxidant capacity of water-soluble (ACW) and lipid-soluble (ACL) antioxidants, the MDA content, and the fatty acid composition. A comprehensive breakdown of these results is shown in Table 2.

### 2.2. Temperature Treatments

The birds in the TN room were reared under a controlled ambient temperature and humidity throughout the experiment, following the recommendations for Ross 308 broilers [28]. Identical conditions were maintained in the TN and HS rooms until day 22, with the ambient temperatures gradually being reduced from 32 °C in the first three days to 22 °C on day 22. From day 22 onwards, a modified temperature regime was introduced in the HS room to create cyclic HS conditions, while the temperature in the TN room gradually decreased to 20 °C by the end of the experiment. During the HS phase, the birds were exposed daily to the following temperatures: 24 ± 0.5 °C for 12 h per day (baseline), from 24 ± 0.5 °C to 34 ± 1 °C for 2 h per day (warm-up phase), 34 ± 1 °C for 7 h per day (HS phase), and from 34 ± 1 °C to 24 ± 0.5 °C for 3 h per day (cool-down phase). The relative humidity in both rooms was monitored daily and allowed to fluctuate, but never to fall below 45%. To evaluate the effects of the thermal environment on the thermoregulatory status of the broilers, especially during heat treatment, the temperature–humidity index (THI) for the broilers was calculated daily according to the equation of Tao and Xin [29], as follows:

THI = 0.85T_db_ + 0.15T_wb_ (1)

where T_db_ represents the dry-bulb temperature and T_wb_ represents the wet-bulb temperature, which were further calculated following the equation established by Raza et al. [30].

### 2.3. Experimental Procedure, Sample Collection, and Chemical Analyses

The animals were individually marked on the first day and their body weight (BW) was recorded weekly throughout the trial, including on the day of slaughter. To monitor the broiler growth performance, average daily gain (ADG), average daily feed intake (ADFI), and feed conversion ratio (FCR) were documented.

On day 42, 12 birds per group were randomly selected from each environmental room, weighed, and sacrificed by cervical dislocation and bleeding. Organ and carcass weights were recorded, and the carcasses were dissected into breast, legs, wings, back, and abdominal fat, with each part being weighed separately. Breast muscle pH and temperature were measured at 15 min and 24 h postmortem using a Mettler Toledo Seven2Go portable pH meter (Mettler Toledo, Schwerzenbach, Switzerland), and electrical conductivity was measured at 24 h postmortem using the LF-Star (Ingenieurbüro Matthäus, Nobitz, Germany). Drip loss was measured at 48 h postmortem, and breast meat color was assessed over five consecutive days using the Minolta CM-600d colorimeter (Minolta Co., Ltd., Osaka, Japan), as described by Voljč et al. [31]. The right part of the breast muscle was divided into six pieces for evaluation under different storage conditions, with two slices left untreated (fresh meat), two slices kept refrigerated (at 4 °C for 6 days), and two slices kept frozen (at −20 °C for 3 months) and packed in polypropylene plastic bags. All breast meat samples were homogenized with liquid nitrogen and a knife mill (Grindomix GM200, Retsch GmbH and Co., Haan, Germany) and stored at −80 °C prior to analysis.

The concentrations of vitamin E (α- and γ-tocopherol) in the feed and meat samples were determined following the method of Leskovec et al. [32], including the modifications described by Pečjak et al. [33]. For tocopherol analysis, the feed (300 mg) and meat (1.3–1.4 g) samples were homogenized and treated with ethanol before extraction with hexane. The hexane phase was collected and evaporated under nitrogen, and the residues were dissolved in ethanol for analysis. The tocopherol concentrations were then determined by reverse-phase HPLC (Agilent, Santa Clara, CA, USA) using a Prodigy 5 μm ODS2 column (Phenomenex, Torrance, CA, USA) with methanol as the mobile phase at a flow rate of 1.5 mL/min. The selenium content in fresh breast muscle was determined by atomic absorption spectrometry (PerkinElmer Inc., Waltham, MA, USA) according to the European standard EN 14.627, as described by Leskovec et al. [32].

To determine the ACW and ACL in feed samples and the ACW in breast meat samples, photochemiluminiscence was used, following the ACW-Kit and ACL-Kit protocols provided by the manufacturer (Photochem, Analytik Jena, Jena, Germany). The ACL procedure is described in detail by Leskovec et al. [32]. Briefly, to determine the ACW in the feed and breast muscle samples, 0.2 g of homogenized feed and 0.3 g of homogenized breast meat were mixed with 1200 µL and 600 µL of 2% m-phosphoric acid, respectively, on a vortex for 10 min and then centrifuged (20,000× *g* for meat and 15,000× *g* for feed, 10 min, 4 °C). The supernatants were mixed with the following reagents: 1500 µL of R1 (miliQ water), 1000 µL of R2 (buffer), and 25 µL of R3 (luminol), and analyzed.

The MDA levels of the breast meat samples were determined according to the method described by Voljč et al. [31] and Pečjak et al. [33]. Frozen, homogenized meat samples (0.3 g) were mixed with 1.0 mL of 2.5% trichloroacetic acid and 100 µL of butylhydroxytoluene solution. After centrifugation (15,000× *g*, 15 min, 4 °C), the supernatant (0.75 mL) was mixed with 1.5 mL of 0.6% thiobarbituric acid solution and heated at 90 °C for 1 h. After cooling, the samples were filtered and analyzed by HPLC (Agilent, Santa Clara, CA, USA) using a 1260 Infinity FLD fluorescence detector with a mobile phase of 65% KH_2_PO_4_ buffer and 35% methanol at a flow rate of 1.0 mL/min.

The fatty acid composition was determined by gas chromatography, as described by Voljč et al. [10]. Briefly, approximately 0.5 g of homogenized feed and meat samples was transmethylated in situ using 0.5 M NaOH in methanol, followed by 14% BF_3_ in methanol. The fatty acid methyl esters (FAMEs) were extracted with hexane, with C19:0 added as an internal standard prior to esterification. The FAMEs were analyzed using an Agilent 6890 (Agilent, Santa Clara, CA, USA) gas chromatograph equipped with an Omegawax 320 column (30 m × 0.32 mm i.d. × 0.25 μm) (Supleco, Bellefonte, PA, USA) and a flame ionization detector.

### 2.4. Statistical Analyses

To conduct ANOVAs, the MIXED procedure of the SAS software (Ver. 9.4, SAS Institute Inc., Cary, NC, USA) was used. In the statistical model, environmental conditions (E), dietary fat treatments (F), antioxidant supplementation (A), and all possible interactions between them (E × F, E × A, F × A, and E × F × A) were included as the fixed effects, while replication pen was considered as a random effect. For continuous measurements, the storage time was included in the model as a fixed effect. A 3-way ANOVA was run for the ADFI and FCR parameters with the fixed effects being environmental conditions (E), dietary fat treatment (F), antioxidant supplementation (A), and their interactions (E × F, E × A, F × A and E × F × A). Least square means (LSMs) were estimated by applying the LSMEANS statement in the model, and differences between LSMs were evaluated with the Tukey–Kramer multiple comparison test. Dispersion was quantified as the standard error of the mean (SEM). Statistical significance was set at *p* < 0.05.

## 3. Results

### 3.1. Growth Performance and Carcass Characteristics

The inclusion of n-3 PUFAs in the diet (NRC N-3 groups) reduced the final BW of the broilers by 20.5% under TN and by 30.0% under HS compared to the NRC group. In addition, the broilers in the NRC N-3 group had lower ADG and ADFI compared to the HAOX and HAOX N-3 groups, regardless of the environmental conditions (Table 3). High dietary PUFAs with low antioxidant levels (NRC N-3 group) also tended (*p* = 0.058) to worsen FCR (Table 3). Exposure to cyclic HS resulted in a 10.8% decrease in final BW compared to TN, along with decreases in ADG and ADFI, but had no effect on FCR. Antioxidant supplementation led to increases in BW and ADFI and a decrease in FCR. A significant interaction between dietary fat and antioxidant supplementation was found for ADG (*p* = 0.016), with a higher ADG observed when antioxidants were administered without high PUFAs.

As shown in Table 4, the chickens fed with high-PUFA diets (NRC N-3 group) had a lower dressing percentage, breast yield, and abdominal fat yield and a higher wing yield than the chickens in the HAOX group. Birds exposed to HS had a higher dressing percentage and higher leg and wing yield compared to those reared under TN. In contrast, antioxidant supplementation (HAOX group) increased breast yield and abdominal fat yield, while wing yield decreased compared to the NRC N-3 group. Significant interactions were found between dietary fat and antioxidant supplementation for dressing percentage (*p* = 0.019) and between environment and antioxidant supplementation for back yield (*p* = 0.004), with antioxidants improving back yield under TN conditions.

### 3.2. Breast Meat Quality Parameters

High dietary PUFA levels (NRC N-3 groups) decreased pH and increased drip loss compared to the HAOX groups in both environments. Conversely, HS had no effect on the pH, drip loss, or electrical conductivity of the breast muscle (Table 5). However, broilers reared under HS conditions had a higher T_15min_ in their breast muscle, which cooled faster after slaughter compared to those reared under TN conditions. In addition, a significant interaction between environment and antioxidant supplementation was found for pH_15min_ (*p* = 0.014) and electrical conductivity (*p* = 0.007). The combination of HS and low antioxidant levels decreased pH_15min_, while high antioxidant levels reduced electrical conductivity under HS.

High dietary PUFA levels (NRC N-3 group) increased the lightness (L*) and decreased the redness (a*) of breast meat during cold storage (*p* < 0.05) compared to the HAOX group, with an interaction between dietary fat and antioxidant supplementation showing a higher L* value at 48 h (*p* = 0.035) and 72 h (*p* = 0.011) when antioxidant levels were low (Table 6). HS had no effect on breast meat color, except after 96 h, where a* values were lower under HS than under TN conditions. A significant interaction between environment and dietary treatment (*p* = 0.005) showed that both stressors increased L* values after 72 h of storage. Antioxidant supplementation decreased a* and b* values (*p* < 0.01) throughout storage, with interactions showing lower a* values in broilers supplemented with high antioxidant levels (HAOX N-3 groups) at 48 h (*p* = 0.042) and 72 h (*p* = 0.011) compared to those supplemented with low antioxidant levels, regardless of their rearing environment.

### 3.3. Oxidative Stability of Breast Meat

Dietary n-3 PUFA enrichment (NRC N-3 group) had a negative effect on the oxidative stability of the stored breast meat, as indicated by a higher MDA content compared to the other groups, especially under TN conditions (Table 7). However, neither the environmental conditions nor the storage process affected the tocopherol and ACW contents in the differently stored breast meat. Antioxidant supplementation significantly increased the tocopherol, selenium, and ACW contents and decreased the MDA levels in breast meat, especially in diets without high PUFA levels (HAOX group). Significant interactions indicated that lower dietary PUFA levels combined with antioxidant supplementation increased the α-tocopherol content in chilled breast meat, particularly under TN conditions (*p* < 0.05). Conversely, this trend was reversed for the γ-tocopherol concentration under different storage conditions (*p* < 0.001). In addition, the storage process significantly affected the MDA content in the NRC (*p* = 0.011) and NRC N-3 (*p* = 0.002) groups reared under TN conditions.

### 3.4. Fatty Acid Composition of Breast Muscle

Table 8 shows the fatty acid profile of the fresh breast meat, which varied according to the different dietary fat sources. As expected, broilers fed with high levels of n-3 PUFAs (NRC N-3 and HAOX N-3 groups) had a higher PUFA content in their breast meat, particularly due to a higher content of LNA (C18:3n-3) and a lower n-6/n-3 PUFA ratio compared to the non-supplemented groups (NRC and HAOX groups, respectively). A significant interaction between environment and dietary fat treatment (*p* < 0.05) was observed, with higher levels of C16:0, C18:1, C18:2n-6, C20:4n-6, MUFA, SFAs, and n-6 PUFAs in the breast meat of birds fed with a more saturated diet (NRC group) under HS conditions. Supplementation with high antioxidant levels resulted in lower total PUFA, n-3 PUFA, and n-6 PUFA contents in breast meat compared to groups fed with the low-antioxidant diet.

## 4. Discussion

Broiler meat production is susceptible to various external stressors, including environmental and nutritional factors. It has been previously reported that oxidized diets [3,6,31] and HS [34] induce the overproduction of ROS and cause oxidative stress, which negatively affects chicken health and production and deteriorates meat quality. However, several nutritional strategies have been found to be effective in mitigating the negative effects of oxidative stress, with vitamin E, vitamin C, and selenium supplementation showing positive effects on the oxidative stability of broiler meat [20,22,35]. However, the NRC [25] nutrient requirements do not consider the additional supplementation of vitamin E and selenium under stress conditions. In contrast, the Aviagen practical nutritional specifications [26] include higher doses of vitamin E (55–80 IU/kg) and selenium (0.3 mg/kg) and recommend additional vitamin C supplementation when animals are exposed to high ambient temperatures. The present study, therefore, investigated the changes in various meat quality parameters in broilers exposed to a high n-3 PUFA intake and heat-induced oxidative stress, considering additional antioxidant supplementation.

### 4.1. Growth Performance and Carcass Characteristics

In the present study, both stressors had a negative effect on performance parameters and carcass yields. The current results indicate that a high PUFA intake led to decreases in final BW and ADFI and an increase in FCR compared to non-PUFA-enriched diets. The latter could have been due to the susceptibility of PUFA-rich vegetable oils to oxidative deterioration, leading to accelerated lipid oxidation and even the formation of toxic compounds that may negatively affect broiler performance parameters [36]. However, other studies have reported that a high intake of n-3 PUFAs does not negatively affect broiler performance [4,9,32]. In the present study, HS decreased final BW and ADFI compared to broilers reared under TN conditions, as previously shown in other studies where heat-stressed birds had a lower FI and, consequently, a lower ADG and ADFI and higher FCR, resulting in lower growth [12,20]. Despite the expectation for synergistic negative effects exerted by a high n-3 PUFA intake and HS, no significant interaction was found. This is consistent with the results of Goo et al. [37], where HS and a high stocking density had no interactive effect on broiler performance. High antioxidant supplementation (HAOX group) improved overall performance, which is consistent with previous studies reporting the protective effects of vitamins E and C and selenium on broiler performance under HS [20,23]. Our results confirm that antioxidant supplementation is necessary to counteract the negative effects of a high n-3 PUFA intake and cyclic HS and to ensure optimal growth performance.

In the present study, a high dietary n-3 PUFA intake reduced dressing percentage, breast yield, and abdominal fat yield, which is consistent with the findings of Zuidhof et al. [38], who reported that high levels of flaxseed significantly reduced carcass weight and breast yield. An increased PUFA intake has been associated with reduced fat deposition, likely due to alterations in lipid metabolism, such as enhanced fatty acid β-oxidation, rather than reduced fatty acid synthesis [4]. Birds exposed to HS had a higher dressing percentage and higher leg and wing yields than birds reared under TN conditions. This is in agreement with Teyssier et al. [12], who also reported a higher carcass yield and higher leg and wing yields and attributed this effect to the composition of oxidative fibers in the leg muscles. These fibers are less affected by the reduced FI and glycogen depletion induced by HS than the glycolytic fibers in the pectoralis major. Although HS is usually associated with enhanced fat deposition as an adaptive mechanism to reduce basal metabolism and store more dietary energy as fat [13], our results showed a tendency (*p* = 0.061) for a lower abdominal fat yield under HS compared to TN conditions. Antioxidant supplementation increased breast, back, and abdominal fat yields, which is consistent with our performance results. In contrast, Habibian et al. [20] reported that vitamin E and Se had no effect on carcass, breast, and thigh yields, although vitamin E significantly reduced the abdominal fat yield in broilers exposed to HS.

### 4.2. Breast Meat Quality Parameters

Meat color, especially L* value, and ultimate pH are commonly used indicators of meat quality, as both are easy to measure and correlate with other important traits such as drip loss, texture, and cooking loss [14]. In this study, a high intake of n-3 PUFAs significantly decreased the ultimate pH and redness (a*) of the breast meat, while drip loss and lightness (L*) increased. These changes resulted in the breast meat exhibiting pale, soft, and exudative (PSE)-like characteristics, as previously noted by Betti et al. [6]. The rapid drop in pH observed may be partially attributed to accelerated postmortem glycolysis, as Zhang et al. [3] reported a reduced activity of the sarcoplasmic reticulum Ca2+-ATPase (SERCA) of the breast muscle when oxidized oils were included in the diet. While HS had no effect on breast meat quality, breast muscle temperature was higher at 15 min postmortem than under TN conditions. PSE-like traits were found to be more pronounced with a high n-3 PUFA intake, although this condition is typically associated with pre-slaughter HS due to increased lactic acid production while muscle temperature remains elevated [39]. In contrast to our results, previous studies have shown that in both acute [40] and chronic HS [34], drip loss and lightness increased, while pH and shear force decreased.

High antioxidant supplementation helped to maintain higher pH_15min_ values, reduced electrical conductivity under HS, and decreased redness (a*) and yellowness (b*) throughout storage compared to low-antioxidant diets. Similar to our results, dietary supplementation with vitamin E [41] and Se [42] has shown a protective effect on the sensory properties of meat, as indicated by increased a* and b* values and decreased L* values, as well as an improved shear force and WHC of broiler breast meat. In contrast, other studies have found that vitamins E and C and selenium had no effect on the meat quality traits of broilers under dietary stress and HS [22,43]. These results suggest that high linseed oil supplementation may increase the susceptibility of meat to oxidation processes, which accelerates postmortem glycolysis and contributes to PSE-like characteristics. However, high antioxidant supplementation could attenuate this effect. In addition, the lack of negative impacts of cyclic HS on meat quality could be attributed to possible adaptive mechanisms that allow birds to recover from cyclic HS during cooler periods of the day.

### 4.3. Oxidative Stability of Breast Meat

External stressors increase the production of ROS in the mitochondrial membranes of broiler skeletal muscle, which react with PUFAs and cause lipid peroxidation [8]. The postmortem process accelerates the degradation of endogenous antioxidants in muscles, reducing the shelf life of meat during refrigerated storage, which can be prolonged by dietary antioxidant supplementation [44]. In this study, high linseed oil supplementation increased the MDA content in stored meat, especially under TN conditions. The negative effects of feeding broilers with high levels of linseed (oil, meal) on lipid peroxidation were previously confirmed, with a longer feeding duration impairing the oxidative stability of breast meat [6,10]. A possible explanation for the higher MDA levels could be due to the higher proportion of unsaturated fatty acids in broiler meat, especially LNA, which is more susceptible to oxidation. Higher MDA levels were also observed during prolonged meat storage at 4 °C [45], which partially agrees with our results, where MDA levels increased in the NRC and NRC N-3 groups under TN during chilled storage. Nevertheless, the recommended levels of thiobarbituric acid reactive substances (TBARSs) for an acceptable meat quality range from 0.2 to 0.5 mg/kg [46], with some recommendations allowing values up to <3 mg/kg [45], suggesting that the MDA levels found in the breast meat in the present study reflect a low level of lipid oxidation.

On the other hand, no effects of HS on tocopherol, ACW, or MDA levels were found, which is consistent with our previous findings [33]. In contrast, Lu et al. [34] reported that HS increased the ROS formation and MDA content and reduced the total antioxidant capacity and superoxide dismutase (SOD) activity in the breast muscles of broilers. Although we expected that the combination of both stressors would have even more profound negative effects on oxidation in breast muscle, no interaction was observed between a high dietary n-3 PUFA intake and HS. This aligns with the study by Goo et al. [37], in which no negative interaction between a high stocking density and HS was observed. In contrast, a previous study by Rezar et al. [24] showed a significant negative interaction between cyclic HS and a high PUFA intake, which led to increased MDA levels in the liver.

Dietary antioxidants such as vitamins E and C and selenium improve broiler meat quality under oxidative stress by strengthening the antioxidant defense system, reducing lipid oxidation, and preserving meat color, texture, and overall oxidative stability [47]. In the present study, high antioxidant supplementation increased the levels of tocopherols, ACW, and selenium and decreased the MDA levels in breast meat, especially in broilers fed with PUFA-enriched diets. Previous studies [31,32] have shown that dietary supplementation with vitamin E resulted in higher levels of α-tocopherols and lower levels of γ-tocopherol in the breast muscles of broilers, which is consistent with our results and may be due to a possible alteration in vitamin E metabolism. Vitamin E has also been reported to prevent oxidative processes in broiler meat during storage and heat treatment in broilers fed with high-n-3-PUFA diets [10] or exposed to HS conditions [48]. In the present study, higher levels of water-soluble antioxidants were found in the fresh and frozen stored meat of the HAOX group compared to the NRC N-3 group under TN conditions, suggesting an enhanced antioxidant capacity of the breast meat, possibly due to a sparing effect of vitamin E. In contrast, our previous results showed no differences in the ACW content of breast meat under different stress conditions [22,33]. Consistent with our results, Habibian et al. [20] reported that dietary supplementation with selenomethionine increased the selenium content in breast meat under both TN and HS conditions. In addition, Khan et al. [42] reported that supplementation with 0.3 mg/kg of selenium-enriched probiotics improved the antioxidant system by increasing selenium retention, GPx and SOD activities, and reducing MDA levels in broiler breast meat under HS conditions. Previous studies have also confirmed the synergistic effect of vitamin E and Se [49] and vitamin E and vitamin C [50] on improving the oxidative stability of meat by lowering MDA levels under HS conditions. Overall, our results emphasize the essential role of high antioxidant supplementation in broiler diets to ensure the oxidative stability of meat and meat products, especially under conditions of dietary- and heat-induced oxidative stress.

### 4.4. Fatty Acid Composition of Breast Muscle

The enrichment of feed with a high n-3 PUFA content had a significant impact on PUFA deposition in the tissues of broilers and, thus, on the fatty acid composition of their meat [51]. With higher levels of dietary linseed oil, the n-6/n-3 ratio and SFA and MUFA contents decreased, while the total PUFA and especially the n-3 PUFA contents increased significantly compared to the non-supplemented groups. These findings are consistent with those of Kalakuntla et al. [52], who reported that linseed oil intake increased the LNA, total PUFA, and n-3 PUFA contents and decreased the n-6/n-3 ratio and SFA and n-6 PUFA contents in broiler meat. The combination of cyclic HS and a non-PUFA-enriched diet in this study increased the concentrations of SFAs (palmitic acid) and n-6 PUFAs (linoleic acid and arachidonic acid), which partially supports the findings of El-Tarabany et al. [53], who found that prolonged HS increased the SFA content but decreased the MUFA and PUFA contents in breast and thigh muscle. In the present study, the increase in n-6 PUFA content observed under HS conditions could have been due to a disturbance in the balance of the lipid metabolism, as HS is known to promote fat synthesis and deposition in broiler tissues [34]. In addition, the increased n-6 PUFA content in the breast meat could be attributed to the relatively high content of n-6 PUFAs in the diet compared to linseed-oil-enriched diets.

In this study, antioxidant supplementation resulted in lower total PUFA, n-3 PUFA, and n-6 PUFA contents in breast meat. Similarly, Konieczka et al. [46] reported that the combination of vitamin E and Se supplementation reduced the n-3 PUFA content in thigh meat and the n-6 PUFA content in both breast and thigh meat. However, the authors also observed an increase in the n-3 PUFA content in breast meat when both antioxidants were supplemented. In addition, Voljč et al. [10] reported that supplementation with 200 IU of vitamin E significantly increased the total PUFA, n-3 PUFA, and n-6 PUFA contents in broiler breast meat, while other studies found no effects of vitamin E [54] or vitamin E and Se [55] supplementation on the fatty acid profile of breast meat. The influence of vitamin E and selenium on the fatty acid composition of chicken tissues remains inconsistent. In addition, vitamin E and selenium are known to positively influence the activity of elongases and desaturases, enzymes involved in the synthesis of long-chain PUFAs, which could promote the deposition of PUFAs in tissues [56].

## 5. Conclusions

In conclusion, the results of this study show that a high n-3 PUFA intake and cyclic HS, both individually and in combination, have negative effects on the growth performance, meat quality, and oxidative stability of broiler breast meat. High dietary n-3 PUFA levels decreased dressing percentage and breast yield and negatively affected meat quality parameters, as evidenced by a lower pH, higher drip loss, and altered breast meat color (increased lightness, L*, and decreased redness, a*), resulting in PSE-like meat characteristics. A high n-3 PUFA intake also increased susceptibility to lipid oxidation, as evidenced by the higher MDA levels in the fresh and stored breast meat. Cyclic HS reduced BW and ADFI, but had less impact on meat quality than expected, although increases in leg and wing yields were observed. On the other hand, no profound negative effects of both stressors on meat quality and oxidative stability were observed. These results underline the crucial role of high antioxidant supplementation in counteracting the negative effects of oxidative stress. Supplementation with high levels of vitamin E, vitamin C, and selenium significantly improved the antioxidant status, reduced the lipid peroxidation, and enhanced the oxidative stability of breast meat, especially in broilers fed with n-3-PUFA-enriched diets. These findings suggest that antioxidant supplementation should be considered to maintain meat quality and enhance oxidative stability under conditions of dietary- and heat-induced oxidative stress. However, further studies are needed to evaluate the oxidative stress induced by a high n-3 PUFA intake and HS in broiler production, with particular attention paid to the combined effects on meat quality and oxidative stability. Determining the optimal antioxidant levels for broilers under stressful conditions should also be a priority.

## Figures and Tables

**Table 1:** Composition and calculated nutrient content of the experimental diets fed to broiler chickens during the starter, grower, and finisher periods.

	Starter (d1–10)	Grower (d11–24)	Finisher (d25–42)
Composition of experimental diets ^1^			
Maize (g/kg)	310	380	527
Wheat (g/kg)	140	130	40
Wheat bran (g/kg)	30.0	15.0	0.00
Soya meal (g/kg)	332	280	240
Corn gluten meal (g/kg)	86.0	93.0	94.1
Linseed oil or mixture of animal fats and plant oils (g/kg)	54.0	57.2	57.2
Salt (g/kg)	4.91	4.95	5.05
Monocalcium phosphate (g/kg)	17.1	15.8	14.5
Limestone (g/kg)	14.5	13.4	12.2
L-lysine-HCl (g/kg)	3.50	3.40	3.30
DL-methionine (g/kg)	2.20	1.80	1.50
L-threonine (g/kg)	0.80	0.50	0.30
Mineral-vitamin supplement ^2,3^ (g/kg)	5.00	5.00	5.00
Calculated energy and nutrient contents			
Metabolizable energy (MJ/kg)	12.4	12.8	13.2
Crude protein (g/kg)	246	228	208
Lysine (g/kg)	12.8	11.5	10.3
Methionine (g/kg)	5.97	5.42	4.95
Calcium (g/kg)	9.56	8.75	7.91
Phosphorus—available (g/kg)	4.80	4.42	4.03

Composition and nutrient content of the experimental diets as in Rezar et al. [24]. ^1^ All feed mixtures contained coccidiostat, Maxiban^®^ G160 (Elanco Products Co., Hampshire, UK). ^2^ Calculated to meet the mineral and vitamin requirement for NRC finisher diets or Aviagen finisher diets for Ross 308 broilers. ^3^ In the HAOX and HAOX N-3 experimental diets, Rovimix E50 (DSM, Heerlen, The Netherlands) was used as a source of vitamin E (dl-α-tocopheryl acetate), Rovimix Stay-C35 (DSM, Heerlen, The Netherlands) was used as a source of vitamin C, and SelSaf 3000 (Lesaffre, Marcq en Baroeul, France) was used as an organic Se source (mainly L(+)-selenomethionine).

**Table 2:** Proximate composition, concentration of selenium, contents of α-, γ + β-, and δ-tocopherol, antioxidant capacity of water (ACW)- and lipid (ACL)-soluble compounds, MDA, and the fatty acid composition of the finisher experimental diets.

Component	Dietary Treatments ^1^			
	NRC	NRC N-3	HAOX	HAOX N-3
Dry matter (g/kg)	899	899	898	898
Crude protein (g/kg)	215	214	214	213
Crude fat (g/kg)	84.4	86.7	83.8	84.1
Crude ash (g/kg)	55.0	56.7	58.9	54.8
Crude fiber (g/kg)	68.4	71.2	68.8	75.1
Nitrogen free extract (g/kg)	476	470	472	471
Se (mg/kg)	0.18	0.15	0.39	0.35
Vitamin E (Tocopherol isomers)				
α-tocopherol (mg/kg)	12.2	8.87	250	232
γ + β-tocopherol (mg/kg)	26.8	43.4	27.2	45.1
δ-tocopherol (mg/kg)	5.91	4.39	6.57	5.33
ACW, ACL, MDA				
ACW (mmol/kg)	3.37	2.62	2.95	2.51
ACL (mmol/kg)	0.34	0.25	0.27	0.27
MDA (µmol/kg)	3.81	7.53	3.35	4.40
Fatty acid composition ^2^ (g of fatty acids/100 g of fatty acids)				
C16:0	22.4	8.99	22.1	8.95
∑ C16:1 ^3^	1.41	8.99	1.37	0.16
C18:0	11.0	3.66	10.7	3.61
∑ C18:1 ^3^	32.0	20.0	31.7	20.0
C18:2 n-6	26.5	28.4	27.5	28.5
C18:3 n-3	2.08	37.8	2.09	37.8
Sum of SFA	37.0	13.4	36.3	13.3
Sum of MUFA	34.0	20.4	33.7	20.4
PUFA	29.0	66.2	30.0	66.3
n-3 PUFA	2.14	37.8	2.16	37.8
n-6 PUFA	26.8	28.4	27.7	28.5
n-6/n-3 PUFA	12.5	0.75	12.9	0.76

The content of Table 2 as in Rezar et al. [24]. SFAs = saturated fatty acids; MUFAs = monounsaturated fatty acids; PUFAs = polyunsaturated fatty acids. ^1^ NRC: recommended levels of NRC, no supplementation; NRC N-3: NRC + 5% linseed oil; HAOX: recommended levels of Aviagen for Ross 308 broilers + 200 IU dl-α-tocopheryl acetate + 250 mg vitamin C + 0.15 mg Se/kg feed; and HAOX N-3: HAOX + 5% linseed oil. ^2^ All values are means of two analyses per measured sample. Only prevalent and dietary important fatty acids are listed. ^3^ Sum of isomers.

**Table 3:** Effects of high n-3 PUFA intake, heat stress, and high or low antioxidant supplementation on growth performance of broiler chickens during the experimental period.

Item/Treatment Factor		BW d21 (g)	BW d40 (g)	ADG d21–40 (g/d)	ADFI ^2^ d21–40 (g/d)	FCR ^2^ d21–40 (g/g)
Environment	Diet ^1^					
Thermoneutral (TN)	NRC	746 ^b^	2442 ^ab^	89.3 ^abc^	198 ^abc^	2.49 ^ab^
	NRC N-3	693 ^b^	1942 ^cd^	67.0 ^cd^	181 ^bcd^	3.69 ^a^
	HAOX	958 ^a^	2933 ^a^	106.2 ^a^	211 ^a^	2.10 ^b^
	HAOX N-3	948 ^a^	2830 ^a^	100.3 ^ab^	205 ^ab^	2.25 ^b^
Heat stress (HS)	NRC	777 ^b^	2243 ^bc^	75.7 ^c^	165 ^de^	2.58 ^ab^
	NRC N-3	707 ^b^	1574 ^d^	45.7 ^d^	144 ^e^	3.44 ^ab^
	HAOX	1005 ^a^	2731 ^ab^	89.5 ^abc^	182 ^bcd^	2.34 ^ab^
	HAOX N-3	964 ^a^	2498 ^ab^	80.7 ^bc^	176 ^cd^	2.58 ^ab^
SEM		18.14	111.6	5.401	5.663	0.289
Main effects						
Environment (E)						
TN		864	2537 ^a^	90.7 ^a^	199 ^a^	2.63
HS		836	2262 ^b^	72.9 ^b^	167 ^b^	2.74
*p*-Value		0.060	0.001	<0.0001	<0.0001	0.626
Fat (F) ^3^						
SFA		872 ^a^	2587 ^a^	90.2 ^a^	189 ^a^	2.38 ^b^
N-3 PUFA		828 ^b^	2211 ^b^	73.4 ^b^	177 ^b^	2.99 ^a^
*p*-Value		0.003	<0.0001	<0.0001	0.007	0.009
Antioxidants (A) ^1^						
NRC		731 ^b^	2050 ^b^	69.4 ^b^	172 ^b^	3.05 ^a^
HAOX		969 ^a^	2748 ^a^	94.2 ^a^	194 ^a^	2.32 ^b^
*p*-Value		<0.0001	<0.0001	<0.0001	<0.0001	0.003
*p*-Values interactions						
E × F		0.400	0.347	0.486	0.793	0.769
E × A		0.750	0.916	0.926	0.447	0.381
F × A		0.220	0.010	0.016	0.114	0.058
E × F × A		0.827	0.903	0.751	0.808	0.605

BW = body weight; ADG = average daily gain; ADFI = average feed intake; and FCR = feed conversion ratio. ^1^ Nomenclature of dietary treatments as in Table 2. ^2^ Expressed as an average amount per experimental group, since broilers were housed in pens (3 pens per group). ^3^ The primary fat source in the experimental diets consisted of a mixture of animal fats and plant oils (SFAs) or 5% cold-pressed linseed oil (N-3 PUFAs). ^a–e^ Different superscript letters within the column show significant differences (*p* < 0.05). Mean values are based on 4 birds per replicate and 3 replicates per dietary treatment, *n* = 12.

**Table 4:** Effects of high n-3 PUFA intake, heat stress, and high or low antioxidant supplementation on carcass traits of broiler chickens.

Item/Treatment Factor		DP (%)	Breast Yield (%)	Leg Yield (%)	Wing Yield (%)	Back Yield (%)	AF Yield (%)
Environment	Diet ^1^						
Thermoneutral (TN)	NRC	74.5 ^abc^	30.8 ^ab^	20.7 ^bc^	7.47 ^ab^	14.8	1.38 ^ab^
	NRC N-3	71.9 ^c^	27.7 ^bc^	20.8 ^bc^	7.86 ^ab^	14.8	1.25 ^ab^
	HAOX	75.2 ^ab^	31.1 ^a^	20.3 ^c^	7.18 ^b^	15.6	1.58 ^a^
	HAOX N-3	74.8 ^abc^	29.5 ^abc^	21.5 ^abc^	7.32 ^b^	15.5	1.51 ^a^
Heat stress (HS)	NRC	75.9 ^a^	29.8 ^abc^	22.5 ^a^	7.73 ^ab^	15	1.31 ^ab^
	NRC N-3	72.8 ^bc^	26.7 ^c^	21.9 ^ab^	8.17 ^a^	15.5	0.85 ^b^
	HAOX	76.6 ^a^	31.4 ^a^	21.9 ^ab^	7.48 ^ab^	14.6	1.47 ^a^
	HAOX N-3	75.9 ^a^	29.8 ^abc^	22.4 ^a^	7.66 ^ab^	15.4	1.43 ^a^
SEM		0.656	0.731	0.317	0.16	0.236	0.121
Main effects							
Environment (E)							
TN		74.1 ^b^	29.8	20.8 ^b^	7.76 ^a^	15.2	1.43
HS		75.3 ^a^	29.4	22.2 ^a^	7.45 ^b^	15.2	1.26
*p*-Value		0.013	0.499	<0.0001	0.010	0.971	0.061
Fat (F) ^2^							
SFA		75.5 ^a^	30.7 ^a^	21.4	7.46 ^b^	15.0	1.43 ^a^
N-3 PUFA		73.9 ^b^	28.4 ^b^	21.7	7.75 ^a^	15.3	1.26 ^b^
*p*-Value		0.001	<0.0001	0.197	0.015	0.064	0.048
Antioxidants (A) ^1^							
NRC		75.6 ^a^	28.7 ^b^	21.5	7.80 ^a^	15.0	1.20 ^b^
HAOX		73.8 ^b^	30.4 ^a^	21.5	7.41 ^b^	15.3	1.49 ^a^
*p*-Value		0.0002	0.002	0.887	0.001	0.147	0.001
*p*-Values interactions							
E × F		0.705	0.989	0.107	0.836	0.062	0.412
E × A		0.896	0.204	0.732	0.897	0.004	0.444
F × A		0.019	0.169	0.026	0.269	0.780	0.173
E × F × A		0.933	0.995	0.999	0.995	0.647	0.308

DP = dressing percentage and AF = abdominal fat. ^1^ Nomenclature of dietary treatments as in Table 2. ^2^ The primary fat source in the experimental diets consisted of a mixture of animal fats and plant oils (SFAs) or 5% cold-pressed linseed oil (N-3 PUFAs). ^abc^ Different superscript letters within the column show significant differences (*p* < 0.05). Mean values are based on 4 birds per replicate and 3 replicates per dietary treatment, *n* = 12.

**Table 5:** Effects of high n-3 PUFA intake, heat stress, and high or low antioxidant supplementation on breast meat quality parameters of broiler chickens.

Item/Treatment Factor		pH_15min_	pH_24h_	T_15min_ (°C)	T_24h_ (°C)	Drip Loss (%)	Electrical Conductivity (S/m)
Environment	Diet ^1^						
Thermoneutral (TN)	NRC	6.39 ^bc^	5.92 ^abc^	40.1 ^abc^	8.16 ^ab^	1.09	4.23 ^ab^
	NRC N-3	6.30 ^c^	5.80 ^bc^	40.1 ^bc^	8.65 ^a^	1.45	3.78 ^ab^
	HAOX	6.46 ^ab^	6.05 ^a^	39.9 ^c^	8.26 ^ab^	1.14	4.47 ^ab^
	HAOX N-3	6.37 ^bc^	5.94 ^ab^	40.2 ^abc^	8.01 ^ab^	1.51	3.88 ^ab^
Heat stress (HS)	NRC	6.37 ^bc^	5.85 ^abc^	41.0 ^ab^	7.48 ^b^	1.33	4.37 ^ab^
	NRC N-3	6.28 ^c^	5.71 ^c^	40.8 ^abc^	7.92 ^ab^	1.51	4.85 ^a^
	HAOX	6.53 ^a^	6.04 ^a^	41.0 ^ab^	7.61 ^b^	1.06	4.11 ^ab^
	HAOX N-3	6.48 ^ab^	6.06 ^a^	41.1 ^a^	7.87 ^ab^	1.25	3.30 ^b^
SEM		0.03	0.05	0.24	0.19	0.14	0.26
Main effects							
Environment (E)							
TN		6.38	5.93	40.1 ^b^	8.27 ^a^	1.30	4.09
HS		6.41	5.91	41.0 ^a^	7.72 ^b^	1.29	4.16
*p*-Value		0.120	0.702	<0.0001	0.0003	0.912	0.735
Fat (F) ^2^							
SFA		6.44 ^a^	5.96 ^a^	40.5	7.88	1.16 ^b^	4.29
N-3 PUFA		6.36 ^b^	5.88 ^b^	40.5	8.11	1.43 ^a^	3.95
*p*-Value		0.0003	0.012	0.750	0.104	0.005	0.081
Antioxidants (A) ^1^							
NRC		6.33 ^b^	5.82 ^b^	40.5	8.05	1.35	4.31
HAOX		6.46 ^a^	6.02 ^a^	40.5	7.94	1.24	3.94
*p*-Value		<0.0001	<0.0001	0.740	0.420	0.281	0.058
*p*-Values interactions							
E × F		0.704	0.412	0.641	0.444	0.347	0.360
E × A		0.014	0.056	0.455	0.289	0.095	0.007
F × A		0.692	0.228	0.321	0.117	0.956	0.069
E × F × A		0.646	0.291	0.854	0.339	0.992	0.136

^1^ Nomenclature of dietary treatments as in Table 2. ^2^ The primary fat source in the experimental diets consisted of a mixture of animal fats and plant oils (SFAs) or 5% cold-pressed linseed oil (N-3 PUFAs). ^abc^ Different superscript letters within the column show significant differences (*p* < 0.05). Mean values are based on 4 birds per replicate and 3 replicates per dietary treatment, *n* = 12.

**Table 6:** Effects of high n-3 PUFA intake, heat stress, and high or low antioxidant supplementation on the color characteristics of broiler breast meat during 5 days of cold storage (4 ± 1 °C).

Item/Treatment Factor		L* 24 h	L* 48 h	L* 72 h	L* 96 h	L* 120 h	*p*-Value (Time)	a* 24 h	a* 48 h	a* 72 h	a* 96 h	a* 120 h	*p*-Value (Time)	b* 24 h	b* 48 h	b* 72 h	b* 96 h	b* 120 h	*p*-Value (Time)
Environment	Diet ^1^																		
Thermoneutral (TN)	NRC	55.32 ^abc^	54.94 ^abc^	54.98 ^ab^	54.03 ^ab^	55.21 ^ab^	0.878	3.13 ^abc^	3.38 ^ab^	3.48 ^ab^	3.76 ^ab^	3.45 ^ab^	0.516	20.21	19.86 ^ab^	20.19 ^abc^	20.10 ^abc^	20.87 ^abc^	0.807
	NRC N-3	57.70 ^a^	56.52 ^ab^	55.12 ^ab^	56.70 ^a^	56.74 ^a^	0.311	3.01 ^abc^	3.57 ^a^	3.39 ^ab^	3.43 ^abc^	3.39 ^ab^	0.155	20.07	20.75 ^ab^	19.89 ^abc^	20.63 ^ab^	21.10 ^abc^	0.620
	HAOX	52.84 ^c^	53.36 ^bc^	53.32 ^b^	52.18 ^b^	52.57 ^b^	0.639	2.55 ^bcd^	2.88 ^abc^	3.17 ^abc^	3.41 ^abc^	3.30 ^ab^	0.054	18.83	18.65 ^b^	18.96 ^abc^	19.28 ^abc^	19.42 ^abc^	0.542
	HAOX N-3	54.59 ^abc^	54.70 ^abc^	53.78 ^b^	53.89 ^ab^	53.86 ^ab^	0.603	2.29 ^cd, B^	2.73 ^abc, AB^	2.99 ^abc, A^	2.93 ^bcd, AB^	2.85 ^b, AB^	0.024	18.96	18.57 ^b^	18.63 ^bc^	18.92 ^abc^	19.60 ^abc^	0.212
Heat stress (HS)	NRC	53.80 ^abc^	52.25 ^c^	52.27 ^b^	53.15 ^ab^	53.94 ^ab^	0.690	3.62 ^a^	3.58 ^a^	3.88 ^a^	3.90 ^a^	3.85 ^a^	0.669	20.79	20.45 ^ab^	21.09 ^a^	20.55 ^ab^	21.53 ^a^	0.757
	NRC N-3	57.20 ^ab^	57.40 ^a^	57.18 ^a^	56.25 ^a^	57.03 ^a^	0.965	3.26 ^ab^	3.79 ^a^	3.29 ^ab^	3.19 ^abcd^	3.47 ^ab^	0.252	20.63	21.26 ^a^	20.84 ^ab^	21.06 ^a^	21.30 ^ab^	0.869
	HAOX	53.18 ^bc^	54.63 ^abc^	55.26 ^ab^	53.68 ^ab^	53.68 ^ab^	0.447	2.61 ^bcd^	2.45 ^bc^	2.64 ^bc^	2.86 ^cd^	3.00 ^b^	0.228	19.08	19.28 ^ab^	18.44 ^c^	18.11 ^c^	19.05 ^bc^	0.533
	HAOX N-3	54.58 ^abc^	55.14 ^abc^	54.51 ^ab^	54.48 ^ab^	54.90 ^ab^	0.963	1.98 ^d, B^	2.18 ^c, B^	2.25 ^c, AB^	2.49 ^d, AB^	2.84 ^b, A^	0.001	19.41	18.80 ^b^	18.49 ^c^	18.78 ^bc^	18.84 ^c^	0.789
SEM		1.014	0.757	0.692	0.922	0.855		0.218	0.233	0.213	0.185	0.218		0.6	0.52	0.504	0.508	0.539	
Main effects																			
Environment (E)																			
TN		55.11	54.88	54.30	54.20	54.59		2.74	3.14	3.26	3.38 ^a^	3.25		19.52	19.46	19.42	19.73	20.25	
HS		54.69	54.85	54.80	54.39	54.89		2.87	3.00	3.01	3.11 ^b^	3.29		19.98	19.95	19.72	19.63	20.18	
*p*-Value		0.547	0.962	0.327	0.762	0.629		0.433	0.413	0.110	0.049	0.763		0.283	0.194	0.411	0.762	0.863	
Fat (F) ^2^																			
SFA		53.79 ^b^	53.80 ^b^	53.96 ^b^	55.33 ^a^	53.85 ^b^		2.97 ^a^	3.07	3.29 ^a^	3.48 ^a^	3.40		19.73	19.56	19.67	19.51	20.22	
N-3 PUFA		56.02 ^a^	55.94 ^a^	55.15 ^a^	55.26 ^b^	55.63 ^a^		2.63 ^b^	3.07	2.98 ^b^	3.01 ^b^	3.14		19.77	19.84	19.46	19.85	20.21	
*p*-Value		0.002	0.0004	0.023	0.002	0.004		0.033	0.965	0.040	0.001	0.065		0.927	0.450	0.566	0.341	0.986	
Antioxidants (A) ^1^																			
NRC		56.00 ^a^	55.28	54.89	55.03 ^a^	55.73 ^a^		3.25 ^a^	3.58 ^a^	3.51 ^a^	3.57 ^a^	3.54 ^a^		20.43 ^a^	20.58 ^a^	20.50 ^a^	20.59 ^a^	21.20 ^a^	
HAOX		53.80 ^b^	54.46	54.22	53.56 ^b^	53.75 ^b^		2.35 ^b^	2.56 ^b^	2.76 ^b^	2.92 ^b^	3.00 ^b^		19.07 ^b^	18.83 ^b^	18.63 ^b^	18.77 ^b^	19.23 ^b^	
*p*-Value		0.002	0.155	0.197	0.022	0.002		<0.0001	<0.0001	<0.0001	<0.0001	0.0002		0.002	<0.0001	<0.0001	<0.0001	<0.0001	
*p*-Values interactions																			
E × F		0.811	0.232	0.085	0.851	0.540		0.337	0.886	0.244	0.606	0.966		0.907	0.751	0.758	0.470	0.579	
E × A		0.403	0.125	0.108	0.180	0.202		0.115	0.042	0.011	0.102	0.160		0.797	0.886	0.086	0.125	0.196	
F × A		0.345	0.035	0.011	0.200	0.387		0.508	0.234	0.845	0.714	0.740		0.656	0.137	0.849	0.601	0.991	
E × F × A		0.623	0.057	0.005	0.598	0.503		0.823	0.841	0.617	0.364	0.270		0.894	0.834	0.819	0.452	0.959	

The color of the breast meat is expressed by the values of CIE, where L* = lightness, a* = redness, and b* = yellowness. Color was measured on consecutive days postmortem: 24 h, 48 h, 72 h, 96 h, and 120 h. ^1^ Nomenclature of dietary treatments as in Table 2. ^2^ The primary fat source in the experimental diets consisted of a mixture of animal fats and plant oils (SFAs) or 5% cold-pressed linseed oil (N-3 PUFAs). ^a–d^, ^AB^ Different superscript letters within the row (storage time) and the column indicate significant differences (*p* < 0.05). Mean values are based on 4 birds per replicate and 3 replicates per dietary treatment, *n* = 12.

**Table 7:** Effects of high n-3 PUFA intake, heat stress, and high or low antioxidant supplementation on levels of α- and γ-tocopherol, selenium, ACW, and MDA in fresh, chilled, and frozen stored breast meat.

															*p*-Value for Storage of Breast Meat			
		Fresh Breast Meat					Chilled ^3^ Breast Meat				Frozen Stored ^4^ Breast Meat				α-Toc	γ-Toc	ACW	MDA
Item ^2^/Treatment factor		α-Toc	γ-Toc	ACW	Se	MDA	α-Toc	γ-Toc	ACW	MDA	α-Toc	γ-Toc	ACW	MDA				
Environment	Diet ^1^																	
Thermoneutral (TN)	NRC	305.1 ^c^	152.3	0.198 ^ab^	77.31 ^b^	0.042 ^c^	328.4 ^c^	162.8 ^ab^	0.192	0.083 ^b^	324.3 ^c^	157.6 ^a^	0.189 ^ab^	0.048 ^cd^	0.382	0.735	0.894	0.011
	NRC N-3	230.2 ^c^	170.2	0.152 ^b^	83.19 ^b^	0.086 ^a^	244.2 ^c^	183.4 ^a^	0.155	0.276 ^a^	219.4 ^c^	180.7 ^a^	0.156 ^b^	0.120 ^a^	0.568	0.791	0.973	0.002
	HAOX	2810.6 ^a^	83.6	0.229 ^a^	161.8 ^a^	0.024 ^c^	3129.1 ^a^	99.09 ^cd^	0.209	0.021 ^b^	2931.7 ^a^	92.24 ^bc^	0.228 ^a^	0.021 ^d^	0.221	0.413	0.714	0.333
	HAOX N-3	2280.1 ^ab^	124.1	0.211 ^ab^	162.6 ^a^	0.037 ^c^	2321.9 ^b^	126.6 ^bc^	0.194	0.061 ^b^	2281.3 ^b^	122.6 ^abc^	0.199 ^ab^	0.054 ^bcd^	0.874	0.910	0.750	0.542
Heat stress (HS)	NRC	312.6 ^c^	141.1	0.172 ^ab^	86.23 ^b^	0.034 ^c^	324.2 ^c^	146.4 ^ab^	0.189	0.056 ^b^	325.4 ^c^	147.2 ^ab^	0.203 ^ab^	0.052 ^bcd^	0.669	0.930	0.596	0.430
	NRC N-3	213.2 ^c^	168.0	0.167 ^ab^	76.45 ^b^	0.076 ^ab^	247.0 ^c^	171.1 ^a^	0.17	0.086 ^b^	224.7 ^c^	165.6 ^a^	0.182 ^ab^	0.086 ^ab^	0.519	0.745	0.768	0.443
	HAOX	2463.7 ^ab^	78.79	0.188 ^ab^	170.8 ^a^	0.027 ^c^	2463.2 ^b^	80.44 ^d^	0.188	0.026 ^b^	2451.5 ^ab^	78.94 ^c^	0.209 ^ab^	0.037 ^cd^	0.995	0.977	0.283	0.558
	HAOX N-3	1616.1 ^b^	263.4	0.195 ^ab^	167.3 ^a^	0.049 ^bc^	2407.3 ^b^	143.8 ^ab^	0.22	0.038 ^b^	2533.2 ^ab^	147.6 ^ab^	0.189 ^ab^	0.059 ^bc^	0.253	0.770	0.384	0.968
SEM		243.8	49.09	0.016	6.179	0.005	106.6	9.436	0.016	0.021	137.2	13.19	0.015	0.008				
Main effects																		
Environment (E)																		
TN		1406.5	132.5	0.197	121.2	0.047	1505.9	143.0	0.187	0.110 ^a^	1439.2	138.3	0.193	0.061				
HS		1151.4	162.8	0.181	125.2	0.047	1360.4	135.4	0.192	0.052 ^b^	1383.7	134.8	0.196	0.059				
*p*-Value		0.149	0.389	0.155	0.375	0.873	0.061	0.280	0.680	0.001	0.579	0.715	0.822	0.702				
Fat (F) ^5^																		
SFA		1473.0 ^a^	113.9	0.197	124.0	0.032 ^b^	1561.2 ^a^	122.2 ^b^	0.194	0.046 ^b^	1508.2	119.0 ^b^	0.207 ^a^	0.040 ^b^				
N-3 PUFA		1084.9 ^b^	181.4	0.181	122.4	0.062 ^a^	1305.1 ^b^	156.6 ^a^	0.185	0.115 ^a^	1314.7	154.1 ^a^	0.181 ^b^	0.080 ^a^				
*p*-Value		0.031	0.059	0.181	0.710	<0.0001	0.002	<0.0001	0.396	0.0001	0.058	0.0006	0.018	<0.0001				
Antioxidants (A) ^1^																		
NRC		265.3 ^b^	157.9	0.172 ^b^	80.8 ^b^	0.059 ^a^	286.0 ^b^	165.9 ^a^	0.176 ^b^	0.125 ^a^	273.5 ^b^	162.8 ^a^	0.183 ^b^	0.077 ^a^				
HAOX		2292.6 ^a^	137.5	0.206 ^a^	165.6 ^a^	0.034 ^b^	2580.4 ^a^	112.5 ^b^	0.203 ^a^	0.036 ^b^	2549.4 ^a^	110.3 ^b^	0.206 ^a^	0.043 ^b^				
*p*-Value		<0.0001	0.561	0.006	<0.0001	<0.0001	<0.0001	<0.0001	0.027	<0.0001	<0.0001	<0.0001	0.031	<0.0001				
*p*-Values interactions																		
E × F		0.625	0.277	0.167	0.264	0.708	0.016	0.153	0.166	0.005	0.071	0.375	0.615	0.040				
E × A		0.157	0.294	0.320	0.519	0.059	0.062	0.328	0.894	0.004	0.557	0.327	0.108	0.027				
F × A		0.090	0.202	0.390	0.944	0.006	0.025	0.105	0.121	0.010	0.365	0.133	0.927	0.029				
E × F × A		0.675	0.337	0.741	0.522	0.500	0.018	0.256	0.525	0.042	0.074	0.258	0.938	0.275				

Toc = tocopherol; ACW = antioxidant capacity of water-soluble antioxidants; and MDA = malondialdehyde. ^1^ Nomenclature of dietary treatments as in Table 2. ^2^ α- and γ-tocopherols and expressed in µg/100 g, ACW expressed in µmol ascorbic acid/100 g and MDA expressed in mg/kg. ^3^ Breast meat stored in the refrigerator at 4 °C for 6 days. ^4^ Breast meat stored in the freezer at −20 °C for 3 months. ^5^ The primary fat source in the experimental diets consisted of a mixture of animal fats and plant oils (SFAs) or 5% cold-pressed linseed oil (N-3 PUFAs). ^a–d^ Different superscript letters within the column show significant differences (*p* < 0.05). Mean values are based on 4 birds per replicate and 3 replicates per dietary treatment, *n* = 12.

**Table 8:** Fatty acid profile of fresh breast meat.

		Fatty Acids (mg of Fatty Acids/100 g of Breast Meat) ^2^																
Item/Treatment Factor		C16:0	∑ C16:1 ^3^	C18:0	∑ C18:1 ^3^	C18:2 n-6	C18:3 n-3	C20:4 n-6	C20:5 n-3	C22:4 n-6	C22:5 n-3	C22:6 n-3	SFA	MUFA	PUFA	n-3 PUFA	n-6 PUFA	n-6:n-3 PUFA
Environment	Diet ^1^																	
Thermoneutral (TN)	NRC	416.9 ^b^	86.22 ^ab^	207.5 ^ab^	734.3 ^ab^	394.6 ^ab^	32.93 ^b^	65.19 ^b^	3.136 ^b^	16.69 ^a^	11.38 ^b^	7.71 ^b^	891.4 ^ab^	833.7 ^ab^	638.6 ^cd^	57.24 ^b^	450.7 ^b^	10.01 ^a^
	NRC N-3	351.6 ^b^	49.25 ^bc^	161.6 ^ab^	539.1 ^b^	512.3 ^ab^	590.8 ^a^	29.68 ^c^	30.36 ^a^	2.761 ^b^	43.69 ^a^	19.44 ^a^	551.4 ^bc^	601.0 ^b^	1264.6 ^a^	704.1 ^a^	561.5 ^ab^	0.80 ^b^
	HAOX	394.3 ^b^	78.84 ^ab^	144.5 ^b^	627.0 ^ab^	339.2 ^b^	22.01 ^b^	65.70 ^b^	3.335 ^b^	17.87 ^a^	11.56 ^b^	7.929 ^b^	600.4 ^bc^	716.7 ^ab^	481.8 ^d^	45.97 ^b^	447.9 ^b^	9.72 ^a^
	HAOX N-3	330.1 ^b^	48.22 ^bc^	139.4 ^b^	487.9 ^b^	399.9 ^ab^	454.6 ^a^	29.68 ^c^	25.89 ^a^	2.809 ^b^	47.66 ^a^	17.41 ^a^	502.8 ^bc^	551.5 ^b^	1005.5 ^abc^	560.3 ^a^	446.6 ^b^	0.80 ^b^
Heat stress (HS)	NRC	687.1 ^a^	98.70 ^a^	228.1 ^a^	1025.5 ^a^	612.7 ^a^	42.33 ^b^	77.80 ^a^	4.376 ^b^	16.03 ^a^	13.78 ^b^	11.35 ^b^	1011.7 ^a^	1171.0 ^a^	799.0 ^bcd^	73.14 ^b^	731.2 ^a^	9.97 ^a^
	NRC N-3	306.1 ^b^	30.71 ^c^	147.1 ^b^	422.1 ^b^	483.9 ^ab^	569.0 ^a^	29.76 ^c^	27.31 ^a^	2.763 ^b^	41.18 ^a^	21.25 ^a^	474.1 ^c^	464.6 ^b^	1199.3 ^ab^	677.3 ^a^	532.5 ^ab^	0.79 ^b^
	HAOX	489.8 ^ab^	83.36 ^ab^	185.4 ^ab^	772.5 ^ab^	456.7 ^ab^	31.47 ^b^	67.15 ^b^	3.234 ^b^	17.35 ^a^	11.91 ^b^	8.297 ^b^	753.4 ^abc^	870.6 ^ab^	613.7 ^cd^	57.10 ^b^	566.4 ^ab^	9.85 ^a^
	HAOX N-3	319.1 ^b^	44.63 ^bc^	139.4 ^b^	474.6 ^b^	385.8 ^b^	410.9 ^a^	28.69 ^c^	26.38 ^a^	2.754 ^b^	45.30 ^a^	19.82 ^a^	496.2 ^bc^	535.0 ^b^	947.6 ^abc^	515.5 ^a^	433.2 ^b^	0.84 ^b^
SEM		61.22	9.667	16.63	93.31	52.12	49.13	1.849	1.16	0.555	1.84	1.298	79.55	109.2	91.05	49.25	58.21	0.218
Main effects																		
Environment (E)																		
TN		373.2 ^b^	65.71	163.3	597.1	411.5 ^b^	273.3	46.91 ^b^	15.68	10.03	28.57	13.12 ^b^	573.6 ^b^	675.7	847.6	341.9	476.7 ^b^	5.335
HS		450.5 ^a^	64.35	175.0	673.7	484.8 ^a^	263.4	50.85 ^a^	15.32	9.724	28.04	15.18 ^a^	683.8 ^a^	760.3	889.9	330.8	565.8 ^a^	5.364
*p*-Value		0.048	0.833	0.330	0.222	0.042	0.765	0.009	0.673	0.442	0.695	0.034	0.029	0.248	0.515	0.749	0.017	0.849
Fat (F) ^4^																		
SFA		497.0 ^a^	86.78 ^a^	191.4 ^a^	789.8 ^a^	450.8	30.35 ^b^	68.96 ^a^	3.520 ^b^	16.98 ^a^	12.16 ^b^	8.821 ^b^	751.3 ^a^	898.0 ^a^	633.3 ^b^	58.36 ^b^	549.1	9.888 ^a^
N-3 PUFA		326.7 ^b^	43.28 ^b^	146.9 ^b^	480.9 ^b^	445.5	506.3 ^a^	28.80 ^b^	27.48 ^a^	2.772 ^b^	44.46 ^a^	19.48 ^a^	506.1 ^b^	538.0 ^b^	1104.3 ^a^	614.3 ^a^	493.5	0.811 ^b^
*p*-Value		0.0001	<0.0001	0.001	<0.0001	0.877	<0.0001	<0.0001	<0.0001	<0.0001	<0.0001	<0.0001	<0.0001	<0.0001	<0.0001	<0.0001	0.123	<0.0001
Antioxidants (A) ^1^																		
NRC		440.4	66.22	186.1 ^a^	680.2	500.9 ^a^	306.9 ^a^	50.61 ^a^	16.29	9.560	27.51	14.94	669.2	767.6	975.4 ^a^	378.0 ^a^	569.0 ^a^	5.393
HAOX		383.3	63.84	152.2 ^b^	590.5	395.4 ^b^	229.7 ^b^	47.15 ^b^	14.71	10.20	29.11	13.36	588.2	668.4	762.2 ^b^	294.7 ^b^	473.6 ^b^	5.306
*p*-Value		0.136	0.713	0.008	0.155	0.005	0.026	0.021	0.070	0.118	0.243	0.098	0.101	0.178	0.003	0.023	0.012	0.560
E × F		0.009	0.137	0.119	0.029	0.011	0.487	0.036	0.278	0.482	0.166	0.954	0.004	0.034	0.118	0.480	0.004	0.922
E × A		0.352	0.796	0.467	0.865	0.533	0.825	0.096	0.515	0.955	0.726	0.470	0.442	0.826	0.935	0.869	0.302	0.715
F × A		0.166	0.174	0.120	0.152	0.995	0.042	0.256	0.194	0.130	0.080	0.865	0.167	0.138	0.516	0.054	0.740	0.456
E × F × A		0.171	0.386	0.902	0.317	0.407	0.912	0.032	0.156	0.898	0.683	0.299	0.14	0.299	0.89	0.924	0.213	0.833

SFAs = saturated fatty acids; MUFAs = monounsaturated fatty acids; and PUFAs = polyunsaturated fatty acids. ^1^ Nomenclature of dietary treatments as in Table 2. ^2^ Values represent the means of 2 analyses per sample. Only the prevalent and dietary important fatty acids are listed, whereas the sum of SFAs, MUFAs, and PUFAs are calculated from all analyzed fatty acids. ^3^ Sum of isomers. ^4^ The primary fat source in the experimental diets consisted of a mixture of animal fats and plant oils (SFAs) or 5% cold-pressed linseed oil (N-3 PUFAs). ^a–d^ Different superscript letters within the column show significant differences (*p* < 0.05). Mean values are based on 4 birds per replicate and 3 replicates per dietary treatment, *n* = 12.

## Data Availability

Data are contained within the article.
